# Visual and Refractive Outcomes of Small-Incision Lenticule Extraction in High Myopia: 5-Year Results

**DOI:** 10.1155/2018/5893126

**Published:** 2018-10-21

**Authors:** Alper Ağca, İhsan Çakır, Beril Tülü Aygün, Dilek Yaşa, Yusuf Yıldırım, Burçin Kepez Yıldız, Ahmet Demirok

**Affiliations:** Beyoğlu Eye Research and Training Hospital, Bereketzade Mah., No. 2, Beyoglu, Istanbul, Turkey

## Abstract

**Purpose:**

To report long-term visual and refractive results of small-incision lenticule extraction (SMILE) in treatment of high myopia.

**Materials and Methods:**

Medical records of patients who underwent SMILE for surgical correction of myopia or myopic astigmatism were retrospectively reviewed. Only patients with a preoperative spherical equivalent of subjective manifest refraction (SE) ≥ 6 D and a postoperative follow-up of 5 years were included in the study. Uncorrected distance visual acuity (UDVA), corrected distance visual acuity (CDVA), and SE were analyzed preoperatively and at 1-, 3-, and 5-year postoperative periods.

**Results:**

Thirty-seven eyes of 37 patients were included in the study. The mean attempted SE was −7.47 ± 1.10 D (range −6.00 to −10.00 D). At the 5-year visit, the mean difference between achieved and attempted SE was −0.43 ± 0.47 (0.50 to −1.25 D). Mean postoperative UDVA and CDVA were 0.20 ± 0.18 and 0.06 ± 0.08 logMAR, respectively. At the 1-year visit, 70% and 97% of the eyes were within ±0.50 D and ±1.00 D of the intended correction. At the 5-year follow-up, 59% and 92% percent of the eyes were within ±0.50 D and ±1.00 D of the intended SE, respectively. At the 5-year visit, the efficacy index was 0.89 ± 0.26 and the safety index was 1.16 ± 0.20. Fifty-four percent of the eyes gained one or more lines of CDVA.

**Conclusion:**

SMILE with an intended correction of up to a spherical equivalent of 10 D is safe and effective. However, there is regression of the refractive effect over extended follow-up.

## 1. Introduction

Small-incision lenticule extraction is a relatively new surgical method for surgical treatment of myopia and myopic astigmatism [1]. In this method, a refractive lenticule is cut in corneal stroma, and it is mechanically removed from a 2 to 3.5 side cut. The procedure has some advantages over laser-in-situ keratomileusis (LASIK) and photorefractive keratectomy (PRK). SMILE does not involve a flap, and the length of the side cut that is used for lenticule extraction is shorter when compared with the side cut of LASIK. Thus, it results in less damage to biomechanical properties and innervation of anterior stroma when compared to LASIK [2, 3]. In contrast to PRK, corneal epithelium and anterior stroma are left intact.

Sekundo et al. reported the early (6 months) refractive outcomes of SMILE in 2011, and the procedure became commercially available in 2012 [1]. Early studies confirmed the efficacy and safety of SMILE in mild, moderate, and high myopia [1, 4–10]. Recently, Blum et al. [11] published 5-year results of the initial cohort of 56 eyes. However, it is the only study with a 5-year follow-up and has been the longest follow-up of SMILE to date. Most of the eyes in that study were mild to moderate myopia. To the best of our knowledge, only two other studies report outcomes of SMILE beyond 3-years and only one of these studies report outcomes in high myopia [12, 13]. Accordingly, there is still a need for studies to evaluate SMILE's efficacy and safety at long-term follow-up periods.

The purpose of this study was to report refractive outcomes and safety of SMILE in high myopia and the stability of refractive results 5 years after surgery.

## 2. Patients and Methods

This study was performed in accordance with the ethical standards of the Declaration of Helsinki, and approval was obtained from the Institutional Review Board. Medical records of the patients who underwent SMILE in Refractive Surgery Center of Beyoğlu Eye Training and Research hospital were retrospectively reviewed. Patients with myopia or myopic astigmatism were included in the study if the spherical equivalent of subjective manifest refraction (SE) was ≥6 diopters (D) and if they had at least 5-year follow-up. Only one eye of each patient was included in the study. A random number table was used to select the eye to be included in the study. Manifest refraction, uncorrected distance visual acuity (UDVA), and corrected distance visual acuity (CDVA) as well as peroperative and postoperative complications were recorded preoperatively and at 1, 3, and 5 years after surgery.

### 2.1. Preoperative and Postoperative Examinations

All the patients underwent the standard rigorous preoperative examination that included subjective manifest refraction, cycloplegic objective refraction using an autorefractometer, UDVA and CDVA measurements, slit-lamp evaluation, and dilated fundus examination. Intraocular pressure was pressure measured by Goldmann applanation tonometry. Corneal topography, dynamic infrared pupillography, ocular wavefront analysis, and corneal wavefront analysis were performed with Sirius corneal topography and abberometry system (Costruzioni Strumenti Oftalmici, Italy).

Postoperatively, the patients were scheduled for routine 1 day, 1 week, 1 month, and 1-year visits. The patients were scheduled for 2 yearly visits thereafter. In postoperative examinations, pupillography, ocular wavefront analysis, and cycloplegic objective refraction were not repeated in postoperative examinations if not indicated by other examination findings. All the other examinations were repeated in postoperative control visits.

### 2.2. Surgical Procedure

The operation was performed with Visumax femtosecond laser platform (Carl Zeiss Meditec AG, Jena, Germany). The spot distance was 3 *µ*m for lamellar cuts and 2 *µ*m for side cuts. The spot energy was set to 140 nJ. The minimum lenticule side cut thickness was set to 15 *µ*m. The lenticule side cut angle was 120, and the optical zone was 6.5 mm.

When the lenticule and side cut had been created, the surgeon positioned the eye under the operating microscope of the laser platform. Under the operating microscope, a blunt spatula was inserted into the anterior lamellar photodisruption plane to perform dissection of any remaining attachments. The same maneuver was performed in the posterior lamellar photodisruption plane. After the lenticule was completely dissected from the overlying and underlying stroma, it was extracted through the side cut with forceps.

Postoperatively, all eyes received dexamethasone eyedrops 4 times daily for 2 weeks and moxifloxacin 0.5% drops 4 times daily for 5 days. Artificial tears were prescribed to be used for at least 1 month.

### 2.3. Statistical Analysis

The data were analyzed with Microsoft Excel 2007 (Microsoft, Inc., Redmond, WA, USA) and PASW Statistics 18 (SPSS, Inc., Chicago, IL, USA). The mean, standard deviation, and frequency were used in the statistical analysis. The normality of the data was confirmed using the Shapiro–Wilk test (*p* < 0.05). Repeated measures analysis of variance (ANOVA) was used to compare preoperative and postoperative visits, and post hoc analyses with Bonferroni corrections were performed when statistically significant differences were detected. Cochran's Q test was used to determine if there were differences on dichotomous dependent variables during follow-up.

The safety index (ratio between postoperative CDVA and preoperative CDVA) in addition to the efficacy index (ratio between postoperative UDVA and preoperative CDVA) were calculated. The data were plotted in sets of six standard graphs that summarized efficacy, predictability, safety, refractive astigmatism, and stability using Microsoft Excel templates.

## 3. Results

Thirty-seven eyes of 37 patients were included in the study. Mean patient age was 31 ± 10 years. Twelve (32%) patients were male, and 18 (68%) were female. The mean preoperative SE was −7.82 ± 1.35 diopters (D) (range 6.00 to −11.00 D). Preoperative characteristics of the eyes are shown in Table 1.

### 3.1. Efficacy

**Figure 1:**
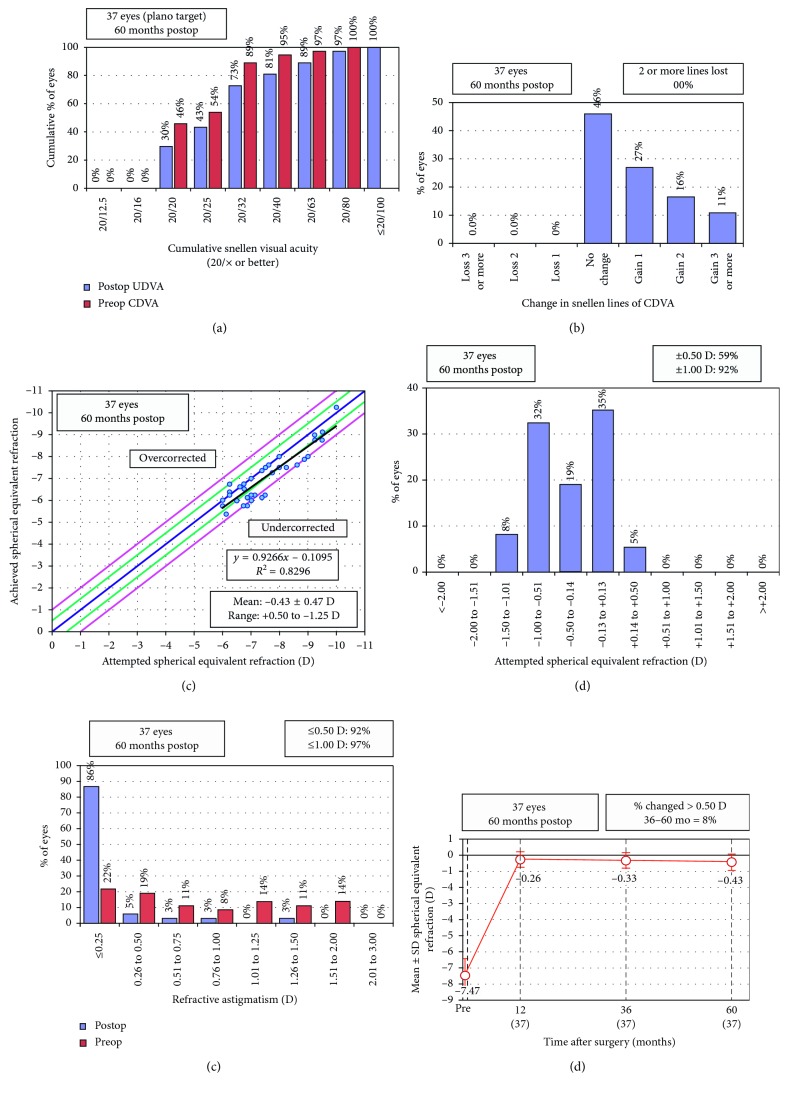
Refractive outcomes at 5 years postoperatively for 37 eyes with high myopia (≥6 D. UDVA: uncorrected distance visual acuity; CDVA: corrected distance visual acuity; D: diopters; Postop: postoperative; Preop: preoperative. (a) Uncorrected distance visual acuity. (b) Change in corrected distance visual acuity. (c) Spherical equivalent attempted vs achieved. (d) Spherical equivalent refractive accuracy. (e) Refractive astigmatism. (f) Stability of spherical equivalent refraction.

Cumulative Snellen visual acuities are shown in Figure 1(a). UDVA, CDVA, and efficacy index during follow-up are shown in Table 2. Preoperative mean UDVA was 1.41 ± 0.18 (ranging from 1.80 to 1.00) logMAR, and preoperative mean CDVA was 0.12 ± 0.12 (ranging from 0.52 to 0) logMAR. At the 5-year follow-up visit, the mean UDVA improved to 0.20 ± 0.18 (ranging from 0.7 to 0) logMAR, and efficacy index (postoperative UDVA/preoperative CDVA) was 0.89 ± 0.26.

### 3.2. Safety

At the 5-year follow-up visit, the mean CDVA was 0.06 ± 0.08 and safety index (postoperative CDVA/preoperative CDVA) was 1.16 ± 0.20. At five years after surgery, no patient had lost two or more lines of CDVA. The change of CDVA lines is shown in Figure 1(b). No vision-threatening complications occurred during surgery or the postoperative period.

### 3.3. Predictability

At the 5-year follow-up, the mean difference between achieved and attempted correction was −0.43 ± 0.47 (0.50 to −1.25 D). The scatterplot of the attempted versus achieved correction of 37 eyes after 5 years is shown in Figure 1(c). At the 1-year follow-up, 70% and 97% of the eyes were within ±0.50 D and ±1.00 D of the intended correction (Table 3). At the 5-year follow-up, 59% and 92% percent of the eyes were within ±0.50 D and ±1.00 D of the intended correction, respectively (Table 3 and Figure 1(d)). However, Cochran's Q test determined that the difference was not statistically significant (Table 3). Ninety-two of the eyes had astigmatism <0.50 D (Figure 1(e)).

### 3.4. Stability

Figure 1(f) shows the difference in SE between intended and achieved correction as a function of time. The difference between intended and achieved corrections was statistically different at postoperative one and three, and five years (repeated measures ANOVA, *p*=0.041). A post hoc analysis revealed that the difference between 1- and 5-year visits were statistically significant (paired samples T-Test, Bonferonni correction, two-tailed *p* value = 0.022). The difference between 1- and 3-year visits (*p*=0.315) and the difference between 3- and 5-year visits (*p*=0.099) were not statistically significant. At the 3-year visit, 92% of patients were within ±0.50 D of the 3-year visit.

## 4. Discussion

The short-term clinical results of SMILE have been quite intensively investigated in recent years. Early reports of SMILE have confirmed its safety and efficacy in surgical treatment of myopia and myopic astigmatism [1, 4–10]. However, long-term refractive outcomes after SMILE are not well established. Accordingly, efficacy, safety, and stability were the main parameters evaluated in this study.

We found that SMILE is an effective and safe procedure for surgical correction of myopia. The efficacy index in our study was 0.93 ± 0.25 at one year. Although this decreased to 0.89 ± 0.26 at 5 years, the difference was not statistically significant. In the literature, the efficacy of SMILE in cases of high myopia was found to be lower when compared to its efficacy in mild to moderate myopia [13–16]. It is well known that the efficacy of LASIK and PRK are also lower in cases of high myopia when compared to their efficacy in mild to moderate myopia [3]. Jin et al. [15] compared early visual results after SMILE in high myopia versus mild to moderate myopia. They found that the efficacy index of SMILE was 0.98 ± 0.18 in high myopia, and it was significantly worse when compared to the efficacy index in mild to moderate myopia. In this study, the efficacy index decreased during follow-up. Also, we found that 27% of the eyes gained two or more lines of CDVA. It is our clinical experience that most patients still have an UDVA equal to or better than the preoperative CDVA, even in the presence of a residual refractive error. The increase in CDVA is probably the reason for the high efficacy index, despite the residual refractive errors and a small but statistically significant regression over time. In other words, the increase in UDVA and CDVA may have masked at least a proportion of decrease in efficacy index over time. In addition to that, there was a trend of increase in the safety index over time (Table 2).

High myopia is known to be a risk factor for long-term regression after laser refractive surgery [17]. A previous study of LASIK reported that the myopic regression for moderate to high myopia was −1.66 ± 2.15 diopters (D) over 15 years, indicating a regression rate of −0.11 D per year [18]. To establish the regression rate after SMILE, a long follow-up period is needed. To the best of our knowledge, there is no study reporting 5-year results after SMILE in cases of high myopia. Xia et al. [19] compared 3-year results between SMILE and LASIK. Although they reported a statistically significant regression in the LASIK group, the mean regression in the SMILE group between 1 year and 3-years visits was only 0.14 D, and it was not statistically significant. We found a trend of regression in our study with achieved SE of −0.26 D, −0.33 D, and 0.43 D at 1, 3, and 5 years, respectively. In line with Xia et al. [19], the difference was not significant at 3 years. However, we found that the regression between 1 and 5 years was statistically significant. Considering the linear change (Figure 1(f)) in mean achieved SE after surgery, it is reasonable to conclude that although regression reaches statistically significant level only after 5 years, it starts in the early postoperative period. Accordingly, studies with a relatively short follow-up may not reveal this mild but statistically significant regression [14, 19].

We found that 70% of the eyes were within 0.5 D and 97% of eyes were within 1.0 D of the attempted correction at 1 year. Our results are comparable with the literature. Qin et al. [16] reported six months results in patients with high myopia. In line with our study months postoperatively, they found that 73% of eyes were within 0.5 D and 97% of eyes were within 1.0 D of the attempted correction. However, despite a clinically mild (statistically significant) regression, we found that only 59% of the eyes were within 0.5 D of the attempted correction at the postoperative 5-year visit. In other words, although the regression was clinically mild, it resulted in a considerable number of patients to be undercorrected by more than 0.50 D in the long term.

Kim et al. [14] suggested that SMILE surgery has a similar predictability, independent of the amount of myopic correction. In contrast, Jin et al. [15] recommended that target correction amount in patients with high myopia should be adjusted to avoid undercorrection and achieve greater satisfaction. To the best of our knowledge, the longest follow-up after SMILE in high myopia cases is reported by Burazovitch et al. [13]. They used a correction factor of the order of 8% of the initial total SE value for the high-myopes, but despite this, there were still some eyes with a final undercorrection. They reported 4-year results and found that the SE tends to shift negatively during the first year and then stabilized up to the fourth year. At four years postoperatively, the mean achieved SE was −0.36 ± 0.28, which is comparable to our results at five years. In this study, only 59% of the eyes were within ±0.50 D of the intended SE at five years. In line with Burazovitch et al. [13] and Jin et al. [15], we believe that the intended correction in high-myopia patients should be revised in our future work, to avoid the undercorrection in high myopia patient.

No sight-threatening complications were observed in this study, and we found that SMILE is a safe procedure for long-term periods with a safety index of 1.16 ± 0.20 at five years, which means that there is an improvement in the mean CDVA. It is reported that visual rehabilitation is slower after SMILE, and recovery may be prolonged in some patients. Accordingly, studies with a longer follow-up have shown better safety parameters [11, 13]. In our study, none of the patients lost 2 or more lines of CDVA and 27% gained two or more lines. In line with our study, Blum et al. reported that no patient had lost two or more lines of visual acuity at five years [11]. However, in contrast to our study, approximately 10% of the patients in that study lost one CDVA line. The use of a different version of Visumax platform (200 hHz) may be the reason since none of the patients in our study had lost one CDVA lines at five years.

The limitations of this study were its retrospective nature and the lack of a control group. In addition to these weaknesses, we must underlie the fact that regression of the refractive effect during a long follow-up may (at least partially) result from an increase in the axial length rather than a true regression at the corneal level. Accordingly, the lack of axial length measurement in postoperative visits is another major weakness of this study. However, this is also related to the retrospective nature of the study, because axial length measurement is not a routine part of our postoperative examinations. However, strict follow-up of the cohort that includes all of the *initial* patients who underwent surgery in our refractive surgery unit is a major strength of this study, as all of the patients who had five-year follow-ups were also examined at 1-, 3-, and 5-year follow-up visits.

In conclusion, our results indicate that SMILE is safe, effective for the correction of high myopia (≥6.00 D) at long-term follow-up for up to five years. However, all the parameters examined in this study indicated a regression of the refractive effect in the long term. We believe that the target correction amount in patients with high myopia should be adjusted to avoid undercorrection and increase long-term patient satisfaction.

## Figures and Tables

**Table 1:** Preoperative characteristics.

Parameter	Mean ± SD (range)
Preoperative SE (D)	−7.47 ± 1.09 (−6.00 to −10.00)
Preoperative astigmatism (D)	0.97 ± 0.80 (0.00 to 4.00)
Preoperative mean keratometry (D)	43.63 ± 1.14 (41.61 to 45.56)
Preoperative thinnest corneal thickness (*µ*m)	544 ± 41 (511 to 586)
Intended maximum lenticule thickness (*µ*m)	137 ± 17 (96 to 166)

SD = standard deviation; SE = spherical equivalent; D = diopters.

**Table 2:** Visual acuities, efficacy index, and safety index during postoperative follow-up.

	1 year (mean ± SD)	3 years (mean ± SD)	5 years (mean ± SD)	*p*
UDVA	0.17 ± 0.15	0.18 ± 0.16	0.20 ± 0.18	0.307
CDVA	0.07 ± 0.10	0.07 ± 0.08	0.06 ± 0.08	0.179
Efficacy index	0.93 ± 0.25	0.93 ± 0.27	0.89 ± 0.26	0.229
Safety index	1.14 ± 0.27	1.14 ± 0.24	1.16 ± 0.20	0.487

UDVA: uncorrected distance visual acuity; CDVA: corrected distance visual acuity; SD: standard deviation. ^*∗*^Repeated measures analysis of variance.

**Table 3:** Predictability of small-incision lenticule extraction over 5 years.

	1 year, *n* (%)	3 years, *n* (%)	5 years, *n* (%)	*p* ^*∗*^
≤±0.50 D	26/37 (70)	24/37 (65)	22/37 (59)	0.223
≤±1.00 D	36/37 (97)	34/37 (92)	34/37 (92)	0.449

*n*: number of patients. ^*∗*^Cochrans's Q Test.

## Data Availability

The datasets used and/or analyzed during the current study are available from the corresponding author on reasonable request.
